# Concurrent chemoradiotherapy with or without cetuximab for stage II to IVb nasopharyngeal carcinoma: a case–control study

**DOI:** 10.1186/s12885-017-3552-6

**Published:** 2017-08-24

**Authors:** Yang Li, Qiu-Yan Chen, Lin-Quan Tang, Li-Ting Liu, Shan-Shan Guo, Ling Guo, Hao-Yuan Mo, Ming-Yuan Chen, Xiang Guo, Ka-Jia Cao, Chao-Nan Qian, Mu-Shen Zeng, Jin-Xin Bei, Jian-Yong Shao, Ying Sun, Jing Tan, Shuai Chen, Jun Ma, Chong Zhao, Hai-Qiang Mai

**Affiliations:** 1 State Key Laboratory of Oncology in South China; Collaborative Innovation Center for Cancer Medicine,Sun Yat-Sen University Cancer Center, Guangzhou, People’s Republic of China; 2 Department of Nasopharyngeal Carcinoma, Sun Yat-Sen University Cancer Center, 651 Dongfeng Road East, Guangzhou, 510060 People’s Republic of China; 3 Department of Molecular Diagnostics, Sun Yat-Sen University Cancer Center, Guangzhou, 510060 China; 4 Department of Radiation Oncology, Sun Yat-Sen University Cancer Center, Guangzhou, 510060 People’s Republic of China

**Keywords:** Cetuximab, Intensity-modulated radiotherapy, Nasopharyngeal carcinoma, Cisplatin, Concurrent chemotherapy, Clinical outcome

## Abstract

**Background:**

This study aimed to evaluate the long-term outcome and toxicities in patients with locoregionally advanced nasopharyngeal carcinoma (NPC) treated by concurrent chemoradiotherapy (CCRT) with/without adding cetuximab.

**Methods:**

A total of 62 patients treated with CCRT plus cetuximab were matched with 124 patients treated with CCRT alone by age, sex, pathological type, T category, N category, disease stage, radiotherapy (RT) technique, Epstein-Barr virus (EBV) DNA levels, and Eastern Cooperative Oncology Group (ECOG). Overall survival (OS), progression-free survival (PFS), locoregional recurrence-free survival (LRFS), and distant metastasis-free survival (DMFS) were assessed using the Kaplan–Meier method and log-rank test. Treatment toxicities were clarified and compared between two groups.

**Results:**

A total of 186 well-balanced stage II to IV NPC patients were retrospectively analyzed (median follow-up, 76 months). Compared to CCRT alone, adding cetuximab resulted in more grade 3 to 4 radiation mucositis (51.6% vs. 23.4%; *P* < 0.001). No differences were found between the CCRT + cetuximab group and the CCRT group in 5-year OS (89.7% vs. 90.7%, *P* = 0.386), 3-year PFS (83.9% vs. 88.7%, *P* = 0.115), the 3-year LRFS (95.0% vs. 96.7%, *P* = 0.695), and the 3-year DMFS (88.4% vs 91.9%, *P* = 0.068). Advanced disease stage was the independent prognostic factor predicting poorer OS and PFS.

**Conclusion:**

Adding cetuximab to CCRT did not significantly improve benefits in survival in stage II to IV NPC and exacerbated acute mucositis and acneiform rash. Further investigations are warranted.

## Background

Nasopharyngeal carcinoma (NPC) is an endemic carcinoma in Southern China and Southeast Asia, especially in the Guangdong province, with an annual incidence of 20–30 per 100,000 population [1–3]. Radiotherapy (RT) is the primary treatment, and several prospective randomized trials and meta-analyses have supported the use of combined radiotherapy and chemotherapy [4–10]. NCCN Clinical Practice Guidelines in Oncology recommended concurrent chemoradiotherapy (CCRT) with or without adjuvant chemotherapy as the standard treatment protocol for stage II–IV NPC [11].

Despite improved treatment modalities and techniques yielding excellent survival outcomes, 20%–30% of patients die of distant and/or local-regional relapse [12]. To improve this result, therapies involving molecular targets such as epidermal growth factor receptor (EGFR) have been studied extensively over the last decade. High levels of EGFR have been observed in 80% of patients with locoregionally advanced NPC and it is associated with poor clinical outcome [13]. Cetuximab, an anti-EGFR antibody, showed a survival benefit in patients with locoregionally advanced head and neck squamous cell carcinoma (HNSCC) when combined with RT [14]. In NPC, a phase II study showed that cetuximab combined with carboplatin demonstrates clinical activity for recurrent or metastatic NPC patients with previous treatment failure with platinum-based therapy [15]. The preliminary report of the ENCORE study demonstrated a promising clinical response in using cetuximab combined with CCRT in NPC [16]. A phase II study conducted by Ma et al. reported that concurrent cetuximab-cisplatin and intensity-modulated radiotherapy in locoregionally advanced NPC was feasible and preliminary survival outcomes compared favorably with historic data [17].

However, currently there are still no randomized trials that have been conducted to directly compare the outcome of CCRT alone versus concomitant cetuximab as a first-line treatment of Stage II to IVb NPC. Therefore, the purpose of this study is to compare the long-term outcome and toxicities of the NPC patients treated by CCRT with or without adding cetuximab used as a matched case-control study.

## Methods

**Fig. 1:**
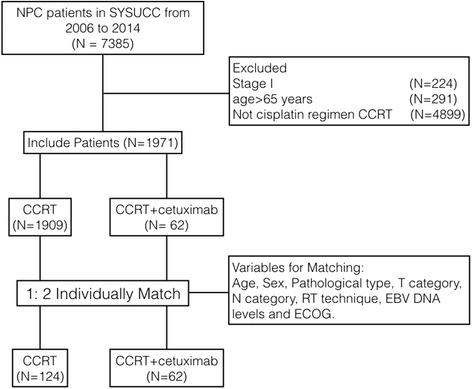
Study flow diagram. NPC, nasopharyngeal carcinoma; SYSUCC, Sun Yat-Sen University Cancer Center; CCRT, concurrent chemoradiotherapy; EBV DNA: Epstein-Barr virus deoxyribonucleic acid; ECOG PS: Eastern Cooperative Oncology Group performance status

Patients diagnosed with nasopharyngeal carcinoma at our institution between January 2007 and April 2014 were identified, as a total of 7385 patients. The eligibility criteria included the following: (1) untreated, newly diagnosed NPC without distant metastasis; (2) biopsy- confirmed World Health Organization (WHO) type II-III NPC; (3) 18 ~ 70 years old; (4) without secondary malignancy, pregnancy, or lactation. Ultimately, 1971 patients were included in the study population, of them 1909 patients received CCRT alone, and only 62 patients received CCRT with cetuximab due to the expensive cost of treatment. In the 1:2 match, patients who received cetuximab plus CCRT were individually matched to two control patients receiving CCRT alone according to age, sex, pathological type, T category, N category, disease stage, RT technique, EBV DNA levels, and ECOG. The inclusion and exclusion criteria are summarized in Fig. 1.

### Pretreatment assessments

All patients were evaluated by a complete physical assessment, hematologic and biochemical profiles, nasopharyngoscopy, MRI or enhanced CT of the nasopharynx and neck (CT was only used in patients with contraindication to MRI), chest scan (X-ray or CT), abdominal sonography, bone scan, and plasma level of EBV DNA. The plasma level of EBV DNA was measured by real-time quantitative polymerase chain reaction (PCR) [18, 19].

### Concurrent chemotherapy and cetuximab

All patients were treated with CCRT using cisplatin chemotherapy. Cisplatin was administered at 80 ~ 100 mg/m^2^ triweekly for at least 2 cycles or at 30 ~ 40 mg/m^2^ weekly for 5 ~ 7 cycles during radiotherapy. In the CCRT group, 64 (51.6%) patients received 80–100 mg/m^2^ cisplatin triweekly and 60 (48.4%) received 30–40 mg/m^2^ cisplatin weekly. In the CCRT plus cetuximab group, 39 (62.9%) patients received triweekly cisplatin, while weekly cisplatin was administered to 23 (37.1%) patients. The patients in the cetuximab plus CCRT group received a loading dose of cetuximab 400 mg/m^2^ 1 week before RT and thereafter a weekly dose of 250 mg/m^2^ during RT for 6 ~ 7 cycles of treatment.

### Radiotherapy

All patients were treated with radiotherapy delivered as five fractions per week, among them, 30 patients underwent conventional RT using two-dimensional technique (2D–CRT) and 156 patients received intensity-modulated radiotherapy (IMRT). Details of the RT techniques applied at our Cancer Center at Sun Yat-Sen University have been reported previously [20, 21] in conformity with the International Commission on Radiation Units and Measurements Reports 50 and 62. The patients treated with 2D–CRT received total radiation doses of 70–76 Gy to the primary tumor at 2 Gy per fraction, 62–66 Gy to the involved areas of the neck, and 50 Gy to the uninvolved areas. The patients received IMRT with variable 2.12 to 2.24 Gy fractions daily for 5 days per week up to a total of 68–72 Gy (median 70 Gy).

### Outcome and follow-up

The primary endpoint for this study was overall survival (OS), which was calculated from the date of treatment to the date of death from any cause. The secondary endpoints for the study were progression-free survival (PFS), distant failure-free survival (DMFS), locoregional failure-free survival (LRFS), and toxicity profile. PFS was calculated from the date of treatment to the date of locoregional failure, distant failure, or death from any cause, whichever occurred first. DMFS was defined as the date of treatment to first distant metastasis, and LRFS was defined as the date of treatment to first locoregional relapse. The Common Terminology Criteria for Adverse Events (CTCAE) version 4.0 was used to grade treatment-related acute toxicities. Acute and late radiation-related complications were scored according to the Radiation Therapy Oncology Group (RTOG)/the European Organization for Research and Treatment of Cancer (EORTC) Late Radiation Morbidity Scoring Schema [22]. All the patients were followed after treatment. Patients were evaluated every 3 months during the first 2 years, and then every 6 months thereafter until death.

### Statistical analysis

Statistical analyses were performed using SPSS software (Version 22.0, SPSS Inc., Chicago, IL, USA). The actuarial survival rates were described with Kaplan–Meier method and survival curves were compared with the log-rank test. Fisher’s exact tests and χ^2^ test were used to assess categorical variables, and hazard ratios (HRs) were estimated using the Cox proportional hazards models. Covariates included in the univariate and multivariate analyses were smoking history, disease stage, EBV DNA level, VCA-IgA, EA-IgA, BMI, C-reactive protein (CRP), and family history of cancer. All statistical tests were two-sided, and the criterion for statistical significance was set at *P* < 0.05.

## Results

### Patient characteristics and treatment compliance

A total of 168 patients were enrolled in this study. There were 62 cases in the cetuximab plus CCRT group and 124 controls. The groups were well matched for age, sex, pathological type, T category, N category, disease stage, RT technique, EBV DNA levels, and ECOG. Patient characteristics and treatment factors are detailed in Table 1. All patients completed planned RT. In terms of chemotherapy, 103 patients received triweekly cisplatin, among whom 39 (62.9%) and 64 (51.6%) patients were from cetuximab plus CCRT group and CCRT group, respectively. The remaining 83 patients received weekly cisplatin, with 23 (37.1%) patients from cetuximab plus CCRT group and 60 (48.4%) patients from CCRT group. In cetuximab plus CCRT group and CCRT group, 60 (96.8%) and 120 (96.8%) patients received at least 2 cycles of triweekly cisplatin or 5 cycles of weekly cisplatin, respectively. Appendix 1: Table 6 lists the specifics of chemotherapy in both groups. The percentages of patients dropping out from treatment due to toxicities were non-significantly different between the two groups. In the group of adding cetuximab, 52 (83.9%) patients received six or more weekly cetuximab doses. 9 (14.5%) patients stopped using cetuximab as a result of toxicity.

**Table 1:** Baseline patient demographic and clinical characteristics

	CCRT with cetuximab group (*n* = 62)	CCRT group (*n* = 124)	*P* value
Age, years			1
Mean	46.32 (25–64)	46.05 (28–66)	
Sex			1
Male	50 (80.6%)	100 (80.6%)	
Female	12 (19.4%)	24 (19.4%)	
Pathological type			1
WHO type II	3 (4.8%)	4 (3.2%)	
WHO type III	59 (95.2%)	120 (96.8%)	
T category			1
T2	14 (22.6%)	28 (22.6%)	
T3	41 (66.1%)	82 (66.1%)	
T4	7 (11.3%)	14 (11.3%)	
N category			1
N0	6 (9.7%)	12 (9.7%)	
N1	25 (40.3%)	50 (40.3%)	
N2	28 (45.2%)	56 (45.2%)	
N3	3 (4.8%)	6 (4.8%)	
Disease stage			1
II	5 (8.1%)	10 (8.1%)	
III	47 (75.8%)	94 (75.8%)	
IVA	7 (11.3%)	14 (11.3%)	
IVB	3 (4.8%)	6 (4.8%)	
RT technique			1
2DRT	10 (16.1%)	20 (16.1%)	
IMRT	52 (83.9%)	104 (83.9%	
Cisplatin delivery			0.144
Every 3 weeks(80–100 mg/m^2^)	39 (62.9%)	64 (51.6%)	
Weekly (30–40 mg/m^2^)	23 (37.1%)	60 (48.4%)	
EBV DNA level			1
< 4000 copies	35 (56.5%)	70 (56.5%)	
≥ 4000 copies	27 (43.5%)	54 (43.5%)	
VCA-IgA			1
< 1:80	15 (24.2%)	30 (24.2%)	
≥ 1:80	47 (75.8%)	94 (75.8%)	
EA-IgA			0.148
< 1:10	24 (38.7%)	35 (28.2%)	
≥ 1:10	38 (61.3%)	89 (71.8%)	
ECOG			1
0	1 (1.6%)	2 (1.6%)	
1	61 (98.4%)	122 (98.4%)	
LDH,U/L			0.666
< 245	61 (98.4%)	120 (96.8%)	
≥ 245	1 (1.6%)	4 (3.2%)	
CRP,g/ml			0.286
< 3.00	49 (79.0%)	89 (71.8%)	
≥ 3.00	13 (21.0%)	35 (28.2%)	
Body mass index, kg/m^2^			1
< 18.5	3 (4.8%)	5 (4.0%)	
≥ 18.5	59 (95.2%)	119 (96.0%)	
Smoking			0.46
Yes	20 (32.2%)	59 (47.6%)	
No	42 (67.7%)	65 (52.4%)	
Family history of cancer			0.02
Yes	13 (21.0%)	11 (8.9%)	
No	49 (79.0%)	113 (91.1%)	

*Abbreviations*: *CCRT* concurrent chemoradiotherapy, *WHO* World Health Organization, *2DRT* two-dimensional radiotherapy, *IMRT* intensity-modulated radiotherapy, *EA* early antigen, *VCA* viral capsid antigen, *IgA* immunoglobulin A, *EBV DNA* Epstein-Barr virus DNA, *ECOG* Eastern Cooperative Oncology Group, *CRP* C-reactive protein, *LDH* serum lactate dehydrogenase levels

### Toxicities

Table 2 lists the distribution of adverse effects. Significant differences only in terms of mucositis were observed between the two treatment groups (51.6% with cetuximab vs. 23.4% without; *P* < 0.001). The rate of 10% weight loss was statistically different (66.1% with cetuximab vs. 50.8% without; *P* = .047). The incidence of cetuximab-related acneiform rash in the cetuximab group was 75.8%. Grade 2 cetuximab-related acneiform rash in the CCRT with cetuximab group was reported in 15 (24.2%) patients. Only one (1.6%) patient developed grade 3 acneiform rash toxic effect and no patient had grade 4 toxic effect. No patient in the CCRT group had acneiform rash. No significant differences of grade 3–4 toxicity in neutropenia, neutropenia, anemia, thrombocytopenia, liver and kidney dysfunction, dermatitis, vomiting, or weight loss were found between the two groups. With respect to late complications, no significant differences of grade 2–4 toxicity in xerostomia and hearing loss were found between the two groups. (Table 3.)

**Table 2:** Cumulative adverse events during treatment by maximum grade per patient during treatment

	Toxic effects, No. (%)				*P* value*
	CCRT alone (*n* = 124)		CCRT + cetuximab(*n* = 62)		
	Grade 1	Grade 2	Grade 1	Grade 2	Grade 1–2
Neutropenia	34 (27.4%)	49 (39.5%)	16 (25.8%)	18 (29.0%)	0.107
Leucopenia	36 (29.0%)	24 (19.4%)	17 (27.4%)	13 (21.0%)	1
Anemia	44 (35.5%)	23 (18.5%)	3 (4.8%)	5 (8.1%)	< 0.001
Thrombocytopenia	19 (15.3%)	10 (8.1%)	5 (8.1%)	3 (4.8%)	0.091
AST increased	17 (13.7%)	3 (2.4%)	9 (14.5%)	1 (1.6%)	1
ALT increased	22 (17.7%)	11 (8.9%)	31 (50.0%)	4 (6.5%)	< 0.001
BUN	9 (7.3%)	1 (0.8%)	2 (3.2%)	1 (1.6%)	0.549
CRE	13 (10.5%)	0	2 (3.2%)	0	0.087
Mucositis	36 (29.0%)	54 (43.5%)	9 (14.5%)	21 (33.9%)	0.001
Dermatitis	73 (58.9%)	26 (21.0%)	28 (45.2%)	23 (37.1%)	0.694
Vomiting	47 (37.9%)	17 (13.7%)	35 (56.5%)	11 (17.7%)	0.003
Weight loss	42 (36.8%)	56 (49.1%)	14 (23.7%)	39 (66.1%)	0.298
Acneiform rash	0 (0.0%)	0 (0.0%)	31 (50.0%)	15 (24.2%)	-
	Toxic effects, No. (%)				*P* value*
	CCRT alone (*n* = 124)		CCRT + cetuximab(*n* = 62)		
	Grade 3	Grade 4	Grade 3	Grade 4	
Neutropenia	18 (14.5%)	1 (0.8%)	8 (12.9)	0 (0.0%)	0.097
Leucopenia	8 (6.5%)	1 (0.8%)	4 (6.5%)	0 (0.0%)	1
Anemia	2 (1.6%)	0 (0.0%)	2 (3.2%)	0 (0.0%)	0.602
Thrombocytopenia	4 (3.2%)	1 (0.8%)	1 (1.6%)	0 (0.0%)	0.665
AST increased	0	0	0	0	-
ALT increased	2 (1.6%)	0	1 (1.6%)	0	1
Renal impairment	0	0	0	0	-
BUN	0	0	0	0	-
CRE	0	0	0	0	-
Mucositis	29 (23.4%)	0 (0.0%)	29 (46.8%)	3 (4.8%)	< 0.001
Dermatitis	5 (4.0%)	0 (0.0%)	3 (4.8%)	0 (0.0%)	1
Vomiting	2 (1.6%)	0 (0.0%)	2 (3.2%)	1 (1.6%)	0.335
Weight loss	0	- ^a^	2 (3.2%)	- ^a^	0.110

Data are n or n (%). *P values were calculated with the χ^2^ test (or Fisher’s exact test). ^a^According to the Common Terminology Criteria for Adverse Events (version 4.0), weight loss has only grade 1–3

*Abbreviations*: *CCRT* concurrent chemoradiotherapy, *AST* aspartate aminotransferase, *ALT* alanine aminotransferase, *BUN* blood urea nitrogen, *CRE* creatinine

**Table 3:** Late toxicities in patients treated with cetuximab + CCRT versus CCRT

Late toxicity	Cetuximab + CCRT (*n* = 62)	CCRT (*n* = 124)	P
Xerostomia*	11 (17.7%)	27 (21.8%)	0.52
Hearing loss*	13 (21.0%)	23 (18.5%)	0.694
Skin dystrophy	3 (4.8%)	5 (4.0%)	1
Neck fibrosis	6 (9.7%)	17 (13.7%)	0.431
Trismus	2 (3.2%)	11 (8.9%)	0.263
Radiation encephalopathy	1 (1.6%)	9 (7.3%)	0.108
Cranial nerve palsy	7 (11.3%)	9 (7.3%)	0.364
*Grade 2–4 toxicities			

*grade 2-4 toxicities

### Survival

**Fig. 2:**
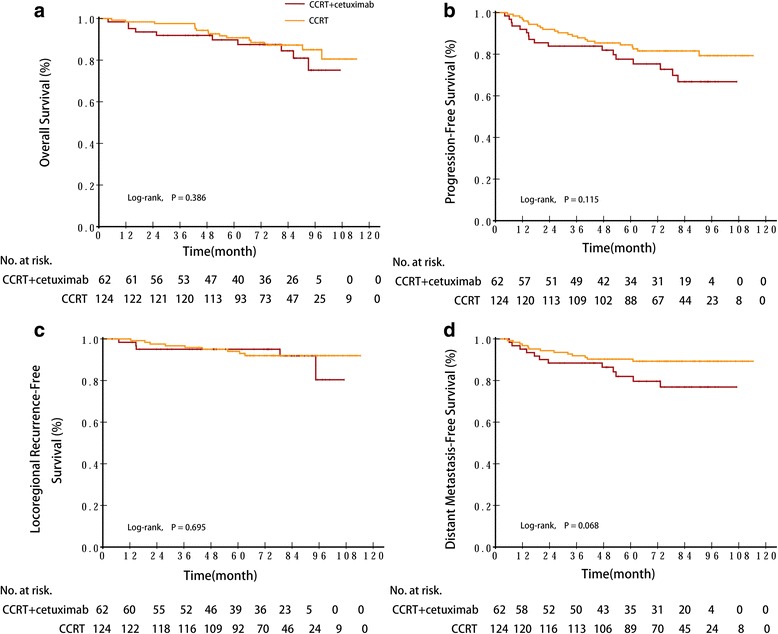
Kaplan–Meier estimates of (**a**) progression-free and (**b**) overall survival and cumulative incidence estimates of (**c**) locoregional failure and (**d**) distant metastasis by assigned treatment. HR, hazard ratio; CCRT, Concurrent Chemoradiotherapy

The median follow-up duration for the entire cohort was 76 months (range, 4–114 months), 76 months (range, 4–107 months) for the cetuximab plus CCRT group, and 76 months (range, 5–114 months) for the controls. No significant differences were found between groups in OS, PFS, LRFS, or DMFS (Table 4, Fig. 2.). The 5-year probabilities for OS were 89.7% (95% CI, 81.9% to 97.5%) for the CCRT with cetuximab group and 90.7% (95% CI, 85.4% to 96.0%) for the CCRT group (*P* = 0.386). The 3-year PFS rates of the CCRT with cetuximab group and the CCRT group were 83.9% (95% CI, 74.7% to 93.1%) and 88.7% (95% CI, 83.0% to 94.4%) (*P* = 0.115), respectively. The 3-year LRFS and DMFS rates of the CCRT with cetuximab group vs. the CCRT group were 95.0% (95% CI, 89.5% to 100%) vs. 96.7% (95% CI, 93.6% to 99.8%) (*P* = 0.695), 88.4% (95% CI, 80.4% to 96.4%) vs. 91.9% (95% CI, 87.0% to 96.8%) (*P* = 0.068), respectively.

**Table 4:** Five years (%) OS (overall survival), PFS (progression-free survival), (distant metastasis-free survival), LRRFS (locoregional relapse-free survival) and HRs with 95% CI

	CCRT plus cetuximab group (%)	CCRT group (%)	Hazard ratio^a^	*P* value
	*N* = 62	*N* = 124	(95% CI)	
Overall survival				
Deaths	6	11	_	
Rate at 5 years	89.7%	90.7%	0.705	0.386
	(81.9–97.5)	(85.4–96.0)	(0.318–1.560)	
Progression-free survival				
Progression	13	19	_	
Rate at 5 years	77.6%	84.5%	0.607	0.115
	(66.6–88.5)	(78.0–91.0)	(0.324–1.137)	
Locoregional relapse-free survival				
Locoregional relapses	3	7	_	
Rate at 5 years	95.0%	94.0%	0.803	0.695
	(89.5–100)	(89.7–98.3)	(0.269–2.403)	
Distant metastasis-free survival				
Distant metastases	10	12	_	
Rate at 5 years	82.0%	90.3%	0.489	0.068
	(71.8–92.2)	(85.0–95.6)	(0.223–1.072)	

Data are n (%) or rate (95% CI). ^a^Hazard ratios were calculated with the unadjusted Cox proportional hazards model. *P* values were calculated with the unadjusted log-rank test

Multiple variables including familial history, smoking history, body mass index (BMI), tumor factors (i.e., disease stage, EBV DNA levels, VCA-IgA, and EA-IgA), and intervention (i.e., whether using cetuximab) were analyzed by a multivariable analysis to predict outcomes for the whole population. Advanced disease stage was an independent prognostic factor predicting poorer OS and PFS (Table 5).

**Table 5:** Cox regression model of multivariable analysis for overall survival and progression-free survival

	HR (95% CI)	*P* value
Overall survival		
Cetuximab (yes vs. no)	1.457 (0.639 ~ 3.322)	0.371
Smoking history (yes vs. no)	1.217 (0.537 ~ 2.757)	0.638
Disease stage (IV vs. II–III)	5.052 (2.194 ~ 11.631)	< 0.001
EBVDNA (≥ vs. < 4000 copies)	2.072 (0.878 ~ 4.893)	0.096
BMI (≥vs. < 23 kg/m^2^)	0.84 (0.373 ~ 1.893)	0.674
CRP (≤ vs. >3.0 g/ml)	0.736 (0.267 ~ 2.027)	0.553
VCA-IgA (< vs. ≥ 1:80)	0.877 (0.209 ~ 3.687)	0.858
EA-IgA (< vs. ≥ 1:10)	0.894 (0.24 ~ 3.326)	0.867
Family history of cancer (yes vs. no)	0.953 (0.271 ~ 3.346)	0.94
Progression-free survival		
Cetuximab (yes vs. no)	1.85 (0.956 ~ 3.58)	0.068
Smoking history (yes vs. no)	0.917 (0.47 ~ 1.79)	0.8
Disease stage (IV vs. II–III)	3.747 (1.823 ~ 7.704)	< 0.001
EBVDNA (≥ vs. < 4000 copies)	1.127 (0.571 ~ 2.222)	0.731
BMI (≥vs. < 23 kg/m^2^)	0.802 (0.424 ~ 1.517)	0.497
CRP (≤ vs. >3.0 g/ml)	1.315 (0.644 ~ 2.688)	0.452
VCA-IgA (< vs. ≥ 1:80)	1.087 (0.376 ~ 3.143)	0.877
EA-IgA (< vs. ≥ 1:10)	0.712 (0.268 ~ 1.892)	0.496
Family history of cancer (yes vs. no)	0.678 (0.233 ~ 1.97)	0.302

*Abbreviations*: *EA* early antigen, *VCA* viral capsid antigen, *IgA* immunoglobulin A, *EBV DNA* Epstein-Barr virus DNA, *CRP* C-reactive protein, *BMI* Body Mass Index

## Discussion

Controversy remains regarding the additional benefit of cetuximab to concomitant chemoradiotherapy, which is the primary regimen for stage II–IV NPC. Appendix 2: Table 7. listed the related studies. Our matched case-control study aimed to clarify the feasibility and efficacy of cetuximab combined with CCRT among stage II–IV NPC patients.

Historically, the treatment of HNSCC with concurrent cetuximab and RT provides survival benefit when compared to RT alone. Bonner et al. conducted a multinational, randomized study to compare radiotherapy alone with radiotherapy plus cetuximab in the treatment of locoregionally advanced squamous-cell carcinoma of the head and neck, which found a survival advantage associated with the use of cetuximab delivered in conjunction with radiation [14]. However, a large randomized phase III trial of Radiation Therapy Oncology Group (RTOG) 0522 [23] in head and neck squamous-cell carcinoma (HNSCC), which tested whether the addition of cetuximab to cisplatin-RT were more effective, demonstrated that no discernable benefit and an increase in toxicity from adding cetuximab to radiation-cisplatin and hence should not be prescribed routinely.

In NPC, to date, studies in terms of cetuximab added to CCRT in NPC have been conducted showing that it is safe, effective, and tolerated [17, 24], while none of them was with a direct comparison of CCRT. An retrospective matched case–control study [25] on concurrent cetuximab-based bioradiotherapy (BRT) or cisplatin-based chemoradiotherapy (CRT) in patients with NPC suggested equivalence between these two treatments. In this study, the 5-year OS rates in patients in the BRT group was similar to patients treated with CRT (79.5% vs. 79.3%, *P* = 0.797). T. Xu et al. earlier reported a randomized phase II study [26] on patients with NPC who received concurrent cetuximab-based radiotherapy (ERT). This study demonstrated that ERT was not more efficacious than concurrent cisplatin-IMRT. In our study, we also disappointed to discover that patients in CCRT + cetuximab group achieved a 5-year OS rate similar to patients treated with re-RT+/−chemotherapy (89.7% vs. 90.7%, *P* = 0.386), and in PFS, LRFS, or DMFS there are also no significant improvements. Survival outcomes in this study seemed much higher than our experience [17, 24, 27] with concurrent cisplatin and radiotherapy—either with or without cetuximab. It may be due to an unbalanced distribution of disease stages in our study, in which the percentages of II, III, and IV stage patients were 8.1%, 75.8%, and 16.1%, respectively. Faced with these negative results, a plausible explanation could be that cetuximab and cisplatin have similar mechanisms of radiation sensitization [28, 29]. So tumors turned out to be resistant to both agents, and sensitive tumors would derive no additional benefit. In further study, refine study populations based on some new biologic tumor features or biomarkers, which were proved to be associated with radiation resistance or metastasis, to define patients who will benefit from cetuximab should be carefully considered.

In regard to toxicity, mucositis is the most common toxicity reported by studies regarding cetuximab combined with radiotherapy in head and neck. Notwithstanding, a pivotal trial by Bonner suggested that cetuximab did not exacerbate mucositis associated with radiotherapy of the head and neck [14]. In daily practice, though not clearly supported in the literature, the rate of mucositis with RT/CRT plus cetuximab seems higher, especially in Asians. Ethnic and lifestyle habits may play a role [30]. A randomized phase II study [26] mentioned before showed using cetuximab with RT was more likely to cause grade 3/4 oral mucositis than cisplatin-based CRT in locally advanced NPC. Ma et al. [17] also reported that using cetuximab with CCRT caused a high rate of grade 3–4 mucositis of 87% in locally advanced NPC. In this study, we found that the incidence of moderate-to-severe mucositis in the CCRT with cetuximab group was significantly higher than that in the CCRT group (51.6% vs. 23.4%, *P* < 0.05). The possible mechanism why cetuximab plus cisplatin add more severe oral mucositis are as followed. The epithelial cells of the oral mucosa are susceptible to the effects of cytotoxic therapy. Cisplatin can interfere with cellular mitosis and reduce the ability of the oral mucosa to regenerate [30], while cetuximab is considered to be able to enhance cytotoxic drug activity. Moreover, as patients in this study received cisplatin in two ways (triweekly and weekly), we have performed a subgroup analysis to rule out the effect of different dose schedule of cisplatin on toxicities. The finding of this stratified analysis showed that cetuximab significantly increased mucositis of NPC patients receiving CRT with triweekly cisplatin, while in weekly cisplatin delivery subgroup, the incidence of grade 3–4 mucositis in CCRT with cetuximab group and CCRT group were 30.4% and 21.7% (*P* = 0.403), respectively (Appendix 3: Table 8). The hematological and other non-hematological adverse events were similar between groups. Ma et al. [17] reported in a single arm retrospective study that the treatment safety was achieved when adding cetuximab to concurrent cisplatin and IMRT in locally advanced NPC. Adding cetuximab and using IMRT were the two prognostic factors predicting severe acute toxicities in this study, while earlier age and 2D–RT were the two prognostic factors predicting severe late toxicities in this study. (Appendix 4: Table 9.)

Multivariable analysis identified stage IV as an independent predictors of poor prognosis. It revealed that adding cetuximab to CCRT was not associated with a lower risk of death and disease progression than CCRT alone. Considering no survival benefit and greater toxicities, cetuximab with CCRT as the first-line treatment should be used with caution and more evidence is needed to guide the use of cetuximab in NPC.

However, there are several limitations to our study. First, the size of our study is relatively small, which might make the results of the study underpowered and selection bias might exist. Second, our study was retrospective and carried out at a single center. Although we tried to decrease potential bias by increasing the numbers in the control group, there is inevitable bias caused by its retrospective nature. Third, we did not rigorously match the delivery method of cisplatin; however, studies [31, 32] have demonstrated that radiation with concurrent cisplatin administered weekly or every 3 weeks leads to similar deliverability, toxicity profiles, and outcomes.

## Conclusion

In conclusion, this study demonstrated that patients with stage II-IV NPC receiving CCRT with cetuximab did not achieve a benefit to survival compared to patients treated with CCRT alone, while adding cetuximab to CCRT exacerbated acute mucositis and acneiform rash. Therefore, multicenter prospective randomized clinical trials with refining study populations are warranted for further investigation.

## Appendix 1

**Table 6:** Chemotherapy details

Cycles	Cetuximab + CCRT (*n* = 62)	CCRT (*n* = 124)
Triweekly regimen		
1	1 (1.6%)	0 (0.0%)
2	28 (45.2%)	50 (40.3%)
3	10 (16.1%)	14 (11.3%)
Weekly regimen		
4	1 (1.6%)	4 (3.2%)
5	4 (6.5%)	16 (12.9%)
6	16 (25.8%)	31 (25%)
7	2 (3.2%)	9 (7.3%)

## Appendix 2

**Table 7:** The list of related studies

Years	Authors	Trial or research	Research design	Primary endpoint	RT/CRT Vs. RT/CRT + cetuximab	*P* value	Cancer
2006	Bonner JA et al.	NCT00004227	RT(*n* = 213) Vs. RT + cetuximab(*n* = 211)	LR control (2 yr)	41% Vs. 50%	0.005	HNSCC
2014	K. Kian Ang et al.	RTOG 0522 Phase III	CRT(*n* = 444) Vs. CRT + cetuximab(*n* = 447)	PFS (3 yr)	61.2% Vs. 58.9%	0.76	HNSCC
2016	Xin Wu et al.	A retrospective matched case–control study	TPF + CRT(*n* = 56) Vs. TPF + RT + cetuximab(*n* = 56)	OS (5 yr)	79.3% Vs. 79.5%	0.797	NPC
2015	T. Xu et al.	NCT01614938 Phase II	CRT(*n* = 23) Vs. RT + cetuximab(*n* = 21)	DFS (3 yr)	78.3% Vs. 85.7%	0.547	NPC

## Appendix 3

**Table 8:** Subgroup analysis based on different dose schedule of cisplatin to compare the toxicities of CCRT and CCRT with cetuximab

	Toxic effects received weekly cisplatin, No. (%)				*p* value*	
	CCRT +Cetuximab(*n* = 23)		CCRT alone(*n* = 60)			
	All Grades	Grade3 ~ 4	All Grades	Grade3 ~ 4	All Grades	Grade3 ~ 4
Leucopenia	14	4	49	11	0.047	1
Neutropenia	13	2	32	5	0.794	1
Anaemia	4	1	36	1	0.001	0.48
Thrombocytopenia	3	1	20	3	0.065	1
AST increased	3	0	10	0	0.945	—
ALT increased	15	1	17	1	0.002	0.48
BUN	0	0	4	0	0.486	—
CRE	0	0	0	0	—	—
Mucositis	23	7	58	13	0.931	0.403
Dermatitis	17	1	50	4	0.507	1
Vomiting	17	2	24	0	0.006	0.074
Weight loss	20	1	54	0	0.996	0.277
	Toxic effects received triweekly cisplatin, No. (%)				*p* value*	
	CCRT +Cetuximab(*n* = 39)		CCRT alone(*n* = 64)			
	All Grades	Grade3 ~ 4	All Grades	Grade3 ~ 4	All Grades	Grade3 ~ 4
Leucopenia	28	4	54	8	0.124	0.978
Neutropenia	21	2	37	4	0.694	1
Anaemia	6	1	33	1	< 0.001	1
Thrombocytopenia	6	0	14	2	0.419	0.525
AST increased	7	0	10	0	0.758	—
ALT increased	21	0	18	1	0.009	1
BUN	3	0	6	0	1	—
CRE	2	0	13	0	0.034	—
Mucositis	39	25	61	16	0.442	< 0.001
Dermatitis	37	2	54	1	0.196	0.66
Vomiting	32	1	42	2	0.072	1
Weight loss	37	1	51	0	0.034	0.379

Data are n or n (%). **p* values were calculated with the χ^2^ test (or Fisher’s exact test). †According to the Common Terminology Criteria for Adverse Events (version 4.0) weight loss has only grade 1–3

## Appendix 4

**Table 9:** Prognostic factors predicting severe acute toxicities

	OR(95% CI)		*P* value
Acute toxicities			
Cetuximab (yes vs. no)	3.011	(1.533–5.915)	0.001
RT technique (IMRT vs. 2D–RT)	8.326	(2.688–25.788)	<0.001
Late toxicities			
Age	0.917	(0.863–0.975)	0.006
RT technique (IMRT vs. 2D–RT)	0.367	(0.133–1.016)	0.054

## Abbreviations

2D–CRT: Two-dimensional conventional radiotherapy
BMI: Body mass index
BRT: Bioradiotherapy
CCRT: Concurrent chemoradiotherapy
CRP: C-reactive protein
DMFS: Distant metastasis-free survival
EBV: Epstein-Barr virus
ECOG: Eastern Cooperative Oncology Group
EGFR: Epidermal growth factor receptor
HNSCC: Head and neck squamous cell carcinoma
HRs: Hazard ratios
IMRT: Intensity-modulated radiotherapy
LRFS: Locoregional recurrence-free survival
NPC: Nasopharyngeal carcinoma
OS: Overall survival
PCR: Polymerase chain reaction
PFS: Progression-free survival
R/M NPC: Recurrent and/or metastatic nasopharyngeal carcinoma
RT: Radiotherapy
RTOG: Radiation Therapy Oncology Group
WHO: World Health Organization
